# Detection of *Brucella* in *Dermacentor* Ticks of Wild Boar with Brucellosis

**DOI:** 10.1155/2024/6618287

**Published:** 2024-01-16

**Authors:** Agustín Rebollada-Merino, Irene Martínez, Clara Duque, Teresa García-Seco, Cristina Escacena, Lucas Domínguez, Antonio Rodríguez-Bertos, Nerea García

**Affiliations:** ^1^VISAVET Health Surveillance Centre, Complutense University of Madrid, Madrid 28040, Spain; ^2^Department of Internal Medicine and Animal Surgery, Faculty of Veterinary Medicine, Complutense University of Madrid, Madrid 28040, Spain; ^3^Department of Population Medicine and Diagnostic Sciences, College of Veterinary Medicine, Cornell University, 240 Farrier Road, Ithaca, NY 14850, USA; ^4^Área de Vigilancia de Riesgos Ambientales en Salud, Consejería de Sanidad, Comunidad de Madrid, Madrid, Spain; ^5^Department of Animal Health, Faculty of Veterinary Medicine, Complutense University of Madrid, Madrid 28040, Spain

## Abstract

Brucellosis is a sanitary and economically relevant disease affecting humans, livestock, and wildlife. Ticks have been suggested as vectors, long-term carriers, and amplifiers of *Brucella*. In this study, ticks from wildlife ungulate hosts living in hunting reserves of a central region of Spain were collected during a 6-year period, pooled, and screened for *Brucella* spp. by PCR. Aiming to correlate *Brucella* spp. DNA presence in ticks with *Brucella* spp. infections in wildlife ungulate hosts, liver samples from deceased wildlife ungulates coming from the hunting reserves showing a positive result for *Brucella* in ticks were tested using a commercial ELISA. In total, 229 tick pools from wild boar (*Sus scrofa*, *n* = 176; 76.8%, 95% CI 70.9%–81.8%), red deer (*Cervus elaphus*, *n* = 40; 17.4%, 95% CI 13.1%–22.9%), mouflon (*Ovis orientalis musimon*, *n* = 7; 3.06%, 95% CI 1.49%–6.17%), and fallow deer (*Dama dama*, *n* = 6; 2.62%, 95% CI 1.21%–5.60%) were analyzed. PCR results showed that 3.93% (95% CI 2.08%–7.30%) tick pools (9/229) from 16.6% hunting reserves (7/41) screened yielded a positive PCR result for *Brucella*. All positive ticks were *Dermacentor (Dermacentor marginatus* or *Dermacentor reticulatus*) collected from wild boar. Ticks collected from wild boars were positive to *Brucella* in a relative percentage of 5.10% (95% CI = 1.61–11.4) in 2018 and of 7.59% (95% CI = 2.79–15.6) in 2021 (6-year prevalence of 5.17%, 9/176). ELISA showed positive results in three wild boars coming from two out of seven hunting reserves (28.5%) with a positive PCR for *Brucella* in ticks. To conclude, *Brucella* spp. DNA can be detected in *Dermacentor* ticks parasitizing wild boars living in hunting reserves harboring *Brucella* spp.-seropositive wild boars. This study provides evidence that the contribution of arthropod vectors should be considered in the epidemiology of brucellosis in wildlife.

## 1. Introduction

Brucellosis (*Brucella* spp.) is a sanitary and economically relevant emerging and reemerging disease affecting humans, livestock, and wildlife worldwide. Thirteen species are recognized in the genus *Brucella* (*B. abortus*, *B. canis*, *B. ceti*, *B. inopinata*, *B. melitensis*, *B. microti*, *B. neotomae*, *B. ovis*, *B. papionis*, *B. pinnipedialis*, *B. suis*, *B. vulpis*, and *B. nosferati*), most of them are zoonotic. *Brucella* species differ from each other according to phenotype, pathogenicity, and host preference [1].

Direct transmission of *Brucella* occurs both vertically or horizontally, and it has been extensively investigated [2]. Vertical transmission occurs transplacental and during delivery. *Brucella* can also be horizontally transmitted via inhalation of aerosolized bacteria, ingestion (milk, unpasteurized products), through mucosal contact with contaminated tissues or its products, and during mating [2, 3]. Indirect transmission of *Brucella* has been less studied. Some *Brucella* spp. (*B. suis*) are supposed to survive in the environment where they may be shed by infected animals and contribute to the transmission on farms [4–6]. Environmental detection of *B. microti*-like during an outbreak at a frog farm has been reported [2]. Recently, it has been shown that the presence of environmental *B. suis* DNA is increased during outbreaks in porcine farms [7].

Rarely, indirect transmission also occurs by means of some blood-feeding arthropods. These may serve as vectors for brucellosis despite their role in transmission is regarded as insignificant in comparison with other routes of infection [2, 8, 9]. Some examples of *Brucella*-transmitting arthropods are bedbugs in human beings (*Cimex* spp.) [10–12], lice in cattle (*Haematopinus tuberculatus*) [13], and ticks [8, 9]. The role of arthropod vectors in the transmission of *Brucella* began to be studied in the mid-20th century. Experimental research showed that *Brucella* spp. could be isolated from ticks (*Rhipicephalus annulatus* and *Amblyomma cajennense*), bedbugs (*Cimex lectularius*), and fleas (*Ctenocephalides felis*), which fed on guinea pigs infected with *B. melitensis*, *B. abortus*, or *B. suis* [14]. Interestingly, among all these arthropods infected with *Brucella*, only ticks were able to transmit the infection to healthy guinea pigs in half of the cases and only if the feed was not interrupted [14]. Later, Russian investigators suggested that *Dermacentor nuttalli* and *Hyalomma marginatum* could disseminate *Brucella* spp. as guinea pigs could be infected by the bite of ticks obtained from cows suffering from brucellosis [15].

More recently, an epidemiological role of ticks in carrying and/or transmitting brucellosis in livestock and pets has been shown. *Brucella* spp. has been identified as *Hyalomma anatolicum*, *D. nuttalli*, and *Dermacentor marginatus* retrieved from cattle and sheep in northeast China [16]. Also, *B. melitensis* has been detected in *Haemaphysalis longicornis* collected from goats or vegetation in central China [17] and in *D. nuttalli* collected from vegetation or sheep in northern China [3, 18]. Similarly, *Brucella* spp. was a prevalent pathogen in *Rhipicephalus sanguineus* parasitizing dogs in Lao PDR [19] and in *Rhipicephalus turanicus* and canine blood samples in North-Western China [20], thus posing owners at risk of contracting brucellosis and raising important public health implications [19, 20]. Furthermore, adult female *D. marginatus* or *D. nuttalli* collected from sheep, cattle, or vegetation in China were demonstrated by both molecular and culture methods to transovarially and transstadially transmit *B. melitensis* and *B. abortus* [3, 21]. Concordantly, *B. melitensis* was more abundant in female adult and larval stages of *D. nuttalli* collected from vegetation or livestock in northern China [3, 18].

The scientific literature suggests ticks as potential vectors, long-term carriers, and amplifiers of *Brucella* spp. However, recent molecular screenings in wild boar (*Sus scrofa*) ticks from Hungary failed to detect *Brucella* spp. despite the high prevalence of *B. suis* in wild boars [22]. Nonetheless, the heterogeneity of brucellosis in different epidemiological settings is well known [23]. Therefore, in this study, we aimed to molecularly screen ticks from wildlife ungulate hosts living in hunting reserves of a central region of Spain during a 6-year period. We anticipate that further serological investigations in deceased wildlife ungulates coming from the hunting reserves showing a positive result for *Brucella* spp. in ticks provide evidence that the contribution of arthropod vectors should be considered in the epidemiology of brucellosis in wildlife.

## 2. Materials and Methods

### 2.1. Permissions

Data were collected as a part of the Research Contract “Analysis for the surveillance and control of zoonoses in wildlife and other infectious agents transmitted by vectors in the Community of Madrid” between the Community of Madrid Health Council and the VISAVET Health Surveillance Centre of the Complutense University of Madrid.

### 2.2. Studied Area

This study was conducted in the Community of Madrid, an 8,028 km^2^ region of central Spain. Livestock farming in the studied area includes caprine (1.53% of the national census), bovine (1.38%), ovine (0.69%), and porcine (0.006%) [24]. Wild ungulates of the studied area are Iberian ibex (*Capra pyrenaica*), fallow deer (*Dama dama*), mouflon (*Ovis orientalis musimon*), red deer (*Cervus elaphus*), roe deer (*Capreolus capreolus*), and wild boar (*S. scrofa*).

### 2.3. Brucellosis in the Studied Area

Spain has been considered free from bovine, ovine, and caprine brucellosis since 2021 [24, 25]. Specifically, the Community of Madrid has been considered free from bovine, ovine, and caprine brucellosis since 2018, and no cases have been reported in the studied area since 2013 [24]. Regarding cases of human brucellosis in Spain, the number of cases studied in the last period was 63 cases in 2017, 40 cases in 2018, 20 cases in 2019, 10 cases in 2020, and 25 cases in 2021 [26]. Specifically, in the Community of Madrid, the number of cases in the period 2016–2018 was six cases, two cases in 2019, two cases in 2020, and two cases in 2021 [27]. Three out of the six human brucellosis cases in the Community of Madrid in the period 2019–2021 have had contact with animals (ranchers) [27].

### 2.4. Samples

Ticks collected from wildlife ungulates (Table 1) living in hunting reserves of the studied area during a 6-year period (2017–2022) and submitted to our laboratory (to be included after acceptance) were included in the study. After collection, ticks were kept in 70% ethanol. Ticks from the same species and geographic area were pooled at a maximum of three ticks per pool according to the tick genera and specie, life stage, sex (adult ticks only), and the individual animal [19]. In case their size was large, they were individually processed.

### 2.5. Tick Identification

Tick specie identification was carried out using the taxonomic key of Estrada-Peña [28] by employing a binocular loupe.

### 2.6. DNA Extraction and *Brucella spp*. PCR

Previously to the extraction, ticks were rinsed in distilled water to wash away traces of ethanol. Then, they were cut as small as possible, and their exoskeleton was crushed. Samples were homogenized in 180 *µ*l of ATL buffer, and DNA was extracted manually using a commercial extraction kit, the DNeasy Blood and Tissue Kit (Qiagen, Hilden, Germany), following the manufacturer's instructions.

*Brucella* detection was performed using a previously described PCR protocol [29]. Positive samples, all of them from wild boar, were assumed to be *B. suis*.

### 2.7. Serology (ELISA)

Deceased wildlife ungulates coming from those hunting reserves showing a positive result for *Brucella* spp. in ticks (PCR) were necropsied, and liver samples were collected for serological studies. To obtain liver transudates for ELISA, liver samples for this purpose were frozen and thawed once at room temperature. Liver transudates were collected and then stored at −40°C until being tested with a commercial ELISA kit, Ingezim Brucella Compact 2.0 (Ingenasa, Madrid, Spain), a multispecies enzymatic assay based on a blocking ELISA technique that uses a monoclonal antibody specific to the epitope C of *Brucella* lipopolysaccharide. Results were interpreted according to the manufacturer's instructions.

### 2.8. Epidemiology Analysis

Individual prevalence was estimated from pool data using the Williams–Moffitt (maximum likelihood) method using WIN PEPI 4.0 software [30].

## 3. Results

### 3.1. Samples Included

In total, 229 tick pools from 41 different hunting reserves (*n* = 4 in 2017, *n* = 6 in 2018, *n* = 3 in 2019, *n* = 6 in 2020, *n* = 16 in 2021, and *n* = 6 in 2022) were screened for *Brucella* DNA.

Tick genera included *D. marginatus* (DM, *n* = 134 pools; 58.5%), *Dermacentor reticulatus* (DR, *n* = 5; 2.18%), *Hyalomma lusitanicum* (HL, *n* = 63; 27.5%), *H. marginatum* (HM, *n* = 2; 0.87%), *Ixodes ricinus* (IR, *n* = 3; 1.31%), *Rhipicephalus bursa* (RB, *n* = 2; 0.87%), and *R. sanguineus* (RS *n* = 20; 12.6%).

Most samples were collected from wild boar (*S. scrofa*, *n* = 176; 76.8%), followed by red deer (*C. elaphus*, *n* = 40; 17.4%), mouflon (*O. musimon*, *n* = 7; 3.05%), and fallow deer (*D. dama*, *n* = 6; 2.62%).

Detailed animal hosts and chronological distribution of samples are shown in Table 1.

### 3.2. *Brucella spp*. PCR in Ticks

Nine over 229 tick pools (3.93%; 95% CI 2.08%–7.30%) from seven different hunting reserves in 2018 and 2021 yielded a positive PCR result for *Brucella* spp. (Table 2). All the ticks in which *Brucella* spp. was detected were collected from different wild boars (*S. scrofa*) individuals and belonged to *Dermacentor* spp. (9/9, 100%), specifically *D. marginatus* (7/9, 77.78%) or *D. reticulatus* (2/9; 22.22%).

The relative percentage of *Brucella* spp. positive samples in the *Dermacentor* spp. pools analyzed was 3.62% (95% CI = 1.76–6.44) for all host species and 3.79% (95% CI = 1.84–6.73) for wild boars. Considering the year in which positive results were obtained, the overall percentage of wild boar tick pools (regardless of the tick genera) with a positive result was 5.10% (95% CI = 1.61–11.4) in 2018 and 7.59% (95% CI = 2.79–15.6) in 2021.

Overall, the percentage of hunting reserves with a positive result for *Brucella* spp. in a tick pool accounted for 16.6% (7/41). Considering the year in which positive results were obtained, the overall percentage of hunting reserves with a positive result was 50.0% (3/6) in 2018 and 25.0% (4/16) in 2021.

### 3.3. Serology (ELISA)

Over the seven hunting reserves (A–G) with a positive result for *Brucella* spp. in a tick pool, liver samples were available in two of them (2/7; 28.5%), D and E, for the year 2021. ELISA showed seropositivity in three wild boar samples from the two hunting reserves studied (2/2; 100%), one from the hunting reserve “D” (1/3; 33.3%), and two from the hunting reserve “E” (2/3; 66.6%) (Table 3).

## 4. Discussion

The wildlife/livestock/human interface in brucellosis is socially and economically complex. Actors related to wildlife are heterogeneous and include hunting and game farming industries, wildlife conservation, and welfare organizations [31]. Recently, the spillover of *B. abortus* from bison (*Bison bison*) and elk (*Cervus canadensis*) to cattle, *B. melitensis* from Alpine ibex (*Capra ibex*) or *B. suis* from wild boar to livestock, hiders eradication efforts and pose at risk Human Health [31, 32]. This is of special concern in countries where brucellosis in cattle, small ruminants, and/or pigs has been eradicated [33] or in countries where control measures for eradication are being implemented. All this highlights the need to investigate brucellosis in wildlife.

Ticks are hematophagous parasites that play a role as vectors and/or reservoirs for many pathogens and are the second most common vector of pathogens after mosquitoes [34]. Herein, we performed a molecular screening of *Brucella* in ticks from wild ungulates (wild boar, red deer, mouflon, and fallow deer) from 41 hunting reserves during a 6-year period. Herein, we molecularly detected *Brucella* in *D. marginatus* or *D. reticulatus* ticks outside Asia. To the best of the authors' knowledge, *Brucella* has not been detected before in wild boar ticks. Previous molecular screenings failed to detect *Brucella* in wild boar ticks from Hungary [22].

In our study, 5.17% of *Dermacentor* ticks collected from wild boar over the 6-year period were positive for *Brucella*. Prevalence of *Brucella* in ticks are highly variable (0%–89%) even within the same country (China): *Brucella* spp. was identified in 0.58% goats and vegetation ticks (*H. longicornis*) [17], 1.32% of vegetation or sheep ticks (*D. nuttalli*) [18], 26.5% cattle and sheep ticks (*H. anatolicum*, *D. nuttalli*, *and D. marginatus*) [16], or 89.0% vegetation or sheep ticks (*D. nuttalli*) [3] depending on the studies. *Brucella* prevalence in dog ticks ranged from 12.4% in Lao PDR (*R. sanguineus*) [19] to 16.74% in China (*R. turanicus*) [20]. The spatial heterogenicity of *Brucella* prevalence in ticks underlines the complexity of brucellosis in the different epidemiological scenarios.

Wild boars are natural hosts and reservoirs for brucellosis and represent an important risk for the reintroduction of the disease into domestic animals [35]. Here, *Brucella* was detected in wild boar ticks but not in ticks from other free-living ungulates studied, such as red deer, mouflon, and fallow deer. Furthermore, the hunting reserves from which *Brucella*-positive ticks were collected yielded seropositive wild boars only. Concordantly, the largest seroprevalence study for *Brucella* in wildlife ruminants (over 13,000 animals) in the Iberian Peninsula over a 10-year period concluded a wild boar seroprevalence of 25%–46% depending on the regions [33]. However, the rest of the wild ungulates studied were not regarded as significant brucellosis reservoirs, including red deer, fallow deer, and mouflon [33]. Concordantly, our results showed that, in wild ungulates other than wild boar, ticks do not seem to play a potential role in *Brucella* transmission. In fact, Spain is regarded as free of brucellosis in small ruminants.

Ticks are suggested as vectors, long-term carriers, and amplifiers of *Brucella*. The experimental vectorial transmission of *Brucella* was proven in guinea pigs some time ago [14, 15]. Here, we could not prove the natural vectorial transmission of *Brucella* in wild boar due to the observational nature of our study. However, studies analyzing canine samples molecularly detected *Brucella* at a detection rate of 16.29% in blood and 16.74% in ticks, demonstrating that ticks carrying *Brucella* are collected from dogs suffering from brucellosis [20]. In our study, a limitation was that we could not prove that individual wild boars parasitized by *Brucella*-carrying ticks were, in fact, infected by *Brucella* due to sample availability. However, we demonstrated that there are *Brucella*-infected wild boars in hunting reserves in which *Brucella*-positive ticks are found parasitizing wild boars, suggesting that ticks should be considered to play a role in the epidemiology of brucellosis in wild boars. By doing so, we employed liver transudates, proven to be an alternative matrix to detect antibodies in many ELISA tests in wild boars [36]. *Brucella* serology likely underestimates the prevalence of infected animals [37, 38], and occasional cross-reactions with other bacteria (*Yersinia enterocolitica*) are found [33], so the combination of bacterial culture and serology should be implemented in subsequent studies.

**Figure 1:**
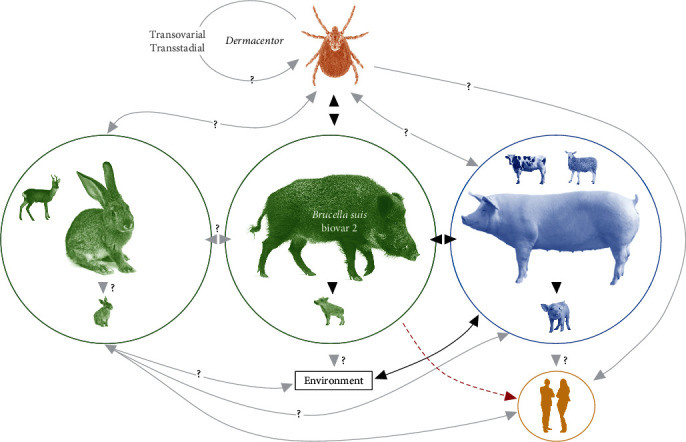
*Brucella suis* biovar 2 transmission. *B. suis* biovar 2 persists in wild boar (*Sus scrofa*) and domestic swine as the primary reservoir hosts, with direct vertical transmission to offspring. Horizontal transmission occurs from wild boar to livestock, humans, and probably other wildlife species. *Dermacentor* ticks are a reservoir for *Brucella suis* biovar 2 and may contribute to its spread among wild boar and potentially other wildlife species, livestock, and humans. Transovarial and transstadial transmission has only been demonstrated for *B. melitensis* and *B. abortus* in *D. marginatus* or *D. nuttalli*. The contribution of the environment in *B. suis* biovar 2 has only been proved for domestic swine.

The scientific literature regards vectorial transmission of brucellosis by ticks as less transcendental than direct transmission [2, 8, 9]. Particularly for *B. suis* biovar 2, the most prevalent species in European wild boar, the role of ticks in the epidemiology is not well known (Figure 1). Anyway, some factors should be considered in the natural vectorial transmission of brucellosis by ticks:

- Presence/abundance of competent vectors parasitizing hosts: ad example, treating cattle for ticks was associated with decreasing risk for brucellosis (*B. abortus*, *B. melitensis*) seropositivity in cattle in a farm- and individual-level [2].
- Ticks as long-term carriers/amplifiers of brucellosis: demonstrated transovarial and transstadial transmission of *Brucella* depending on the tick genera [3, 21].
- A reservoir/vectorial role of ticks in brucellosis: tick parasitization of *Brucella*-infected hosts [14, 15].
- Transmission of *Brucella* through blood: ad example, acquired brucellosis after blood transfusion [39] and minimal infective dose of *Brucella*.
- Endemic brucellosis: maintained direct transmission of *Brucella* among wildlife species [23].
- Host density: ad example, the population of wild boars is dramatically being increased in Europe.

Other contributing factors to be considered in the transmission of any vector-borne disease are climatic factors and landscape structure [40]. Despite the fact that environmental factors were not analyzed here, other authors have pointed out that wild boar density in summer has been proposed as a factor to *Brucella* seropositivity in south-western Spain [41]. In addition, an increased number of arthropods due to climate change has been reported [3].

Our results also suggest the value of ticks for monitor *Brucella* in wildlife, as performed for other vector-borne pathogens. Further studies should monitor the prevalence of brucellosis in ticks in different epidemiological scenarios to improve the understanding of the indirect transmission of *Brucella*. The role of other arthropod vectors (bedbugs, lice, etc.) in the epidemiology of brucellosis should be explored in future research.

Extensive livestock farming in the studied area is residual compared to the rest of Spain, particularly regarding porcine. Thus, potential transmission of *Brucella* from wild boar to domestic pigs, and eventually other domestic ungulates, in the studied area is considered negligible. However, other Spanish regions have abundant porcine extensive farming so further research on *Brucella* transmission risks from wildlife to livestock in other epidemiological scenarios in Spain is warranted.

## Figures and Tables

**Table 1:** Animal host species and tick pools included in the study.

Animal hosts	Ticks							
	Species	2017	2018	2019	2020	2021	2022	Tick pools (%)
Wild boar	DM	3	42	20	6	29	27	127 (72.1)
	DR	3	2	—	—	—	—	5 (2.84)
	HL	7	6	2	6	9	6	36 (20.4)
	HM	—	2	—	—	—	—	2 (1.13)
	IR	—	1	—	1	—	1	3 (1.70)
	RS	—	—	—	—	3	—	3 (1.70)
	Total	13	53	22	13	41	34	176 (100)

Red deer	DM	—	1	—	4	1	—	6 (15.0)
	HL	6	6	3	—	5	—	20 (50.0)
	RB	—	1	—	1	—	—	2 (5.00)
	RS	1	11	—	—	—	—	12 (30.0)
	Total	7	19	3	5	6	0	40 (100)

Mouflon	DM	—	—	—	—	1	—	1 (14.2)
	HL	—	—	—	—	4	—	4 (57.4)
	RS	1	1	—	—	—	—	2 (28.5)
	Total	1	1	0	0	5	0	7 (100)

Fallow deer	HL	—	—	—	—	3	—	3 (50.0)
	RS	3	—	—	—	—	—	3 (50.0)
	Total	3	0	0	0	3	0	6 (100)

All	Total	24	73	25	18	55	34	229
	%	10.4	31.8	10.9	7.86	24.0	14.8	100

**Table 2:** Wild boar (*Sus scrofa*) ticks (*Dermacentor* spp.) positive for *Brucella* spp. (PCR).

Sample	*Dermacentor* spp.	Hunting reserve	Year	Ct value
1	*D. reticulatus*	A	2018	40.68
2	*D. reticulatus*	A	2018	37.23
3	*D. marginatus*	B	2018	35.87
4	*D. marginatus*	C	2018	33.73
5	*D. marginatus*	D	2021	38.19
6	*D. marginatus*	E	2021	36.14
7	*D. marginatus*	E	2021	33.92
8	*D. marginatus*	F	2021	39.17
9	*D. marginatus*	G	2021	34.70

**Table 3:** Serology (ELISA) results from wildlife samples (liver transudate) coming from hunting reserves with wild boar (*Sus scrofa*) ticks (*Dermacentor* spp.) positive for *Brucella* spp. (PCR).

Hunting reserve	Animal species	Seropositive (ELISA)
D	Wild boar	1/3 (33.3%)
D	Fallow deer	0/3 (0.0%)
E	Wild boar	2/3 (66.6%)
E	Red deer	0/1 (0.0%)
E	Fallow deer	0/1 (0.0%)

## Data Availability

Data of this research article are available from the corresponding author upon reasonable request.
